# Family Doctors Seen through the Eyes of Specialists: A Qualitative Study

**DOI:** 10.1155/2013/729473

**Published:** 2013-06-02

**Authors:** Anna Probst, Iris Natanzon, Joachim Szecsenyi, Stefanie Joos

**Affiliations:** ^1^Department of General Practice and Health Services Research, University Hospital Heidelberg, Voßstraße 2, 69115 Heidelberg, Germany; ^2^Competence Center General Practice, Baden-Wuerttemberg, Germany

## Abstract

Germany is facing a shortage of young family doctors. In search of possible reasons the aim of this study was to explore the perception of specialists on family doctors. Within a qualitative study 16 medical specialists from different fields in hospital and outpatient care setting were interviewed. Interviews were analysed using qualitative content analysis according to Mayring. Most of the interviewed specialists have a positive view on family doctors although a certain depreciative assumption is resonated in a number of statements. According to the specialists, family doctors enjoy a high status in public, even if social processes of change may have a negative influence on their rather old-fashioned image. Specialists find that family medicine is underrepresented in university education suffering from an upgrading of specialized disciplines. Altogether the majority of the interviewed specialists certify family doctors in Germany a positive image. Lecturer in medical education and training should be aware of their key role in the career choices of young trainees and avoid degradation or upgrading of certain medical disciplines. Interlinked measures on different levels focusing on the improvement of working conditions and representation at the universities would be needed to regain attractiveness for the family doctor's profession as a career choice for young doctors.

## 1. Introduction

An intact inner-medical cooperation plays an essential role for proper care processes and thus the overall quality of care. However, tensions between different specialist groups are not rare, both in Germany and in other countries. Studies indicate that especially the relationship between family doctors and specialists is often troubled. Studies from Canada show that family doctors do not feel themselves respected enough by their medical professional colleagues [1]. US surveys among students reporting “badmouthing” about family doctors indicate a similar tendency [2]. As of today in Germany there are hardly any studies on whether and how medical professional groups “talk bad” about each other or waste disrespectful remarks towards students about a different group of doctors. In a qualitative study with family doctors some of the interviewees reported about experiences, according to which specialists who work as teacher in a medical faculty or in postgraduate training, convey a negative image of family doctors to students [3, 4].

Not only for reasons of professionalism, but also with respect to the career choices of young physicians who might get influenced by such observations [2, 5], this issue should attract more attention. In Germany patients are not registered with family doctors and, therefore, family doctors compete with neighbour physicians and community specialists for patients. They manage a high patient flow resulting in comparatively short consultations times. In the last years Germany is facing a widespread shortage of physicians which is noticeable already today in some rural regions and which will worsen dramatically in the next 10 years due to the age structure of the still active primary care physicians. A central problem proves to be the shortage of future physicians. The development of the number of doctors shows that fewer and fewer graduates of human medicine opt for a career as family doctor [6]. In addition to processes of social change and unfavorable political conditions also a “bad image” is discussed as a decisive factor for the lack of new recruits to family medicine. Studies show that the image of family medicine conveyed through teachers has got a relevant influence on the career choices of students [2, 7]. However, while the professional self-image of family doctors has already been the subject of scientific investigation [8], there has been little scientific research about the image of family doctors from the perspective of their specialized colleagues. In view of the above the aim of this qualitative study was to explore the image of family doctors from the perspective of specialists.

## 2. Methodology

For the present study, a qualitative approach was chosen in order to obtain a deeper understanding of individual experiences and perspectives of the specialists.

### 2.1. Recruitment of Doctors

A total of 179 specialists in the regions of the city of Karlsruhe, Karlsruhe country, the city of Heidelberg, and the Rhein-Neckar-Kreis were contacted by mail. The selection of these doctors was done randomly using contact addresses given on the website of the Kassenärztliche Vereinigung, Baden-Württemberg. Consideration was given to doctors who had completed a specialist medical training of the following disciplines: internal medicine, surgery, psychiatry, neurology, and radiology. Representatives of these specialist groups were chosen because these specialists maintain direct contact with family doctors. Physicians who were interested in the study were asked to report back by fax: 23 responses were received. 16 out of these 23 responding doctors were selected according to the aim to have a broad sample stratified to the following criteria: female/male, age, working in a hospital or private practice, urban/rural area, University Hospital/peripheral hospital, five different specialties (see above), and duration of the professional activity. After 16 interviews no new aspects were noted assuming that saturation had been reached.

### 2.2. Data Collection and Interview Guide

Semistructured interviews were conducted with the specialists in the respective private practices or hospitals of the doctors. Each interview lasted between 30 and 60 minutes.

Based on a literature review and own preliminary work [3, 4, 8, 9] an interview guideline was created with following four main topics: “own area of expertise,” “own perception of family doctors,” “cooperation between family doctor and specialist,” and “shortage of family doctors.”

The present study addresses the topic of “own perception of family doctors” which was explored by the following key questions.

1. What is your opinion on family doctors? What skills do you think family doctors should have?
2. Which image family doctors have in the public or in society? Did this reputation change over the years?
3. How did you perceive family medicine at university?

### 2.3. Data Preparation and Data Analysis

The interviews were recorded on site using a digital recording device. Afterwards the interviews were transcribed on a pseudonym basis and were analyzed using content analysis according to Mayring [10] with the support of the software program ATLAS.ti (Scientific Software Development GmbH). Selected quotes were combined into different categories. To guarantee the intersubjective traceability according to the quality criteria of qualitative research [11], the categories were discussed among the involved persons of the different disciplines (sociologist, a medical doctor, and medical student) and in case of discrepancies consensus was effected. In addition to the deductive categories derived from the key questions inductive categories were established from the text material. Thus, it was possible to develop a system of categories which was reworked, revised, and supplemented in an iterative process. Subsequently, concise statements (“anchor items”) were selected from all quotes of each category.

## 3. Results

### 3.1. Sample

A total of 16 doctors between the age of 35 and 63 were interviewed. The sample is described in Table 1.

### 3.2. Positive and Negative Image Aspects

The categories “positive image” and “negative image” were derived out of the text material obtained from the answers to the first key question (Table 2).

The majority of specialists respects the work of family doctors and perceives them as important.

*“The majority of them is excellent (…) They care for the patients, they know them (…) actually really good.” (Surgeon 1, hospital).*

They appreciate their good clinical training and also emphasize their farsightedness with regard to the re-assignment of their patients.

*“I've got a number of family doctor colleagues, (…) where I also notice these are people who have gone through a certain sound clinical training, who take good care of their patients and often have a very good diagnostic nose (…), who are very good at deciding which specialists they send the patient to for further evaluation.” (Internist 3, practice).*

Professional aspects of family doctors are highly appreciated and highlighted positively by specialists. In order to react in an adequate and conscientious way, it is important that family doctors have a broad spectrum of knowledge.

*“A family doctor also has got gynecological, urological and orthopedic patients, he must know a bit of neurology. It is quite important to know a little bit from many areas.” (Internist 3, practice).*

One of the key competences family doctors should have in the opinion of the specialists is the function of a coordinator. According to the interviewees family doctors should be responsible to transfer the patients to the right specialists and, where appropriate, to send them for further examinations. When hospitalization is necessary, it is important that the family doctor supplies the colleagues in the hospital with relevant information about the patient.

*“They should hold the threads together I think (…). Ideally, all diagnostic findings should converge with the family doctors and they should also have sufficient expertise and be educated to judge them.” (Internist 2, hospital).*

A third aspect that supports the family doctors positive image in the specialists' opinion is the ability for empathy. According to the experiences of the specialists, family doctors deal with their patients in a very emphatic way. When a patient denies a prescribed therapy, it is also among the tasks of a family doctor to convince him or her of the importance of this therapy.

*“(…) you need to be able to listen, to talk, to convince, you must put yourself in someone, you must be able to bring someone to terms with all that constitutes a human being, with all his strengths and weaknesses (…).” (Radiologist 2, practice).*

For many of the basically positive statements it is striking that there was a negative undertone reflected in specific signal words in the above quotations, such as, for example, “who have gone through a *certain* sound clinical training” or “It is *quite* important to know *a little bit* from many areas.” The same is also reflected by the positive affirmation of basically positive statements, for example,

*“I think that they are universal geniuses, and I literally mean it" (Psychiatrist 3, hospital).*

Many of the interviewed specialists comment on their impression that family doctors seem to be a bit frustrated about their work. For this they hold themselves responsible for the bad working environment and general conditions, which in the opinion of the respondents also have an impact on the quality of patient care.

*“Some [family doctors] heavily complain about the general conditions of society and politics and think that because of these bad conditions and the poor remuneration patients should perhaps (…) not be cared for too well.” (Internist 4, practice).*

Some specialists believe that the primary care profession no longer reflects the current spirit of the time of increasing specialization. Some very few participants deliberately establish the provocative thesis that family doctors “no longer fit into our society.”

*“I think that other aspects which traditionally somehow belong to the occupational image of a family doctor that is some minor surgery, a bit of Gynecology, a bit of Pediatrics, a bit of ENT, I believe, this is a little bit of what no longer fits into our society.” (Internist 3, practice).*

### 3.3. Public Aspects

In this subject area the main categories “public reputation” and “change of patient expectations” were compiled (Table 3).

Many specialists believe that physicians in general and first and foremost family doctors do have a positive reputation in the public.

*“It is still true that the social prestige of the profession as a physician is very high in the population and if you ask lay people, then they associate this high prestige of the medical profession in the first instance (…) with the family doctor, because he is the person who indeed cares for generations of families (…)” (Internist 3, practice).*

A part of the specialists consider the family doctor as politically supported and strengthened.

*“This is being rather well supported, the image. Politically, the family doctor's position is rather strengthened than weakened. Also, in the overall political situation family doctors are more popular than specialists (…).” (Psychiatrist 2, practice).*

Others believe that the public reputation of family doctors has worsened over the past years. It is suggested that one reason for that is that the doctor-patient relationship has changed. The family doctor is no more available round the clock. Changes in practice structures such as the development of large joint practices instead of small single practices and/or the implementation of emergency medical services have started to affect the relationships between patients and their family doctors.

*“No longer be responsible for all day and night, I think that has changed a bit. That means, the gratitude has decreased a little bit, I would say, because in crisis situations, then you are always very grateful if you have been helped.” (Psychiatrist 3, hospital).*

In the opinion of the specialists the reputation of family doctors in the society is strongly influenced by the public media. Often the image of the greedy doctor is being evoked. A considerable part is contributed by policy. Some specialists have the impression that family doctors are more and more downgraded to the function of a “gateway.”

*“In any case degraded, yes, yes. As said, the facts that they are reduced to the gateway function basically and are no longer work as a doctor. Afterwards they only are kind of a point of contact similar to a Toto Lotto office.” (Radiologist 2, practice).*

According to the specialists, one of the reasons for a modified attitude of patients vis-à-vis family doctors is the increasing abundance of diagnostic possibilities which patients claim for themselves. If this is not supported by the family doctor, the patient will consult the next doctor.

*“But patients behave differently (…). They want to have the referral to MRI and that for free and immediately. And that of course is something where the family doctor also gets in trouble, because the patient says: okay, if you do not give me the referral, I go to another doctor.” (Radiologist 2, practice).*

Additionally, many patients look for medical information in the internet which also might influence the doctor-patient relationship. Informed in advance, patients often question the decisions of their doctors more often or directly decide to go to the relevant specialist with their presumed diagnosis.

*“A little bit maybe due to the internet. Through such things patients try to get a little more direct access to the specialist.” (Internist 6, practice).*

Others consider age and multiple offerings in a big town as reasons for patients to directly consult a specialist.

*“In a big town you have many cardiologists, many gastroenterologists and that's why the family doctor is not perceived so necessary as it is the case in rural areas.” (Internist 6, practice).*

*“Yes, the younger patients, 35 years and younger, have a tendency to go directly to the specialists.” (Psychiatrist 1, University Hospital).*

### 3.4. Influence at University

The text material derived from the answers to the third question was the basis for the categories of “representation” and “conveyance of the image” (Table 4).

The image of family medicine among students is perceived very differently by the respondents—partly based on experience from their own studies and partly based on actual experiences with students and medical graduates.

* “Derogative. In our semester family doctors at that time were those people who did not know exactly what they wanted and eventually knew a bit of everything and nothing else.” (Surgeon 1, hospital).*

*“If they start as young fellows here, so directly after graduation, I do not have the impression that they have a bad image of family doctors, no.” (Psychiatrist 3, hospital).*

Many participants report that they have perceived family medicine being underrepresented at the universities. There were frequent statements that either no special activities took place or the offered activities were scheduled at a very late time of day. Some of the specialists became better acquainted with family medicine due to their own initiative during a medical clerkship.

*“(…) I've made one of my first medical clerkships in the practice of a really old country doctor. At that time it was common that during the lunch break, the whole practice team and the family of the doctor sat together at a long table and had lunch. I've still come to know it like that.” (Internist 3, practice).*

Some specialists report negative comments heard during their time at university about family medicine and family doctors and the fact that other disciplines rated themselves higher and acted up.

*“(…), however, its major drawback is that the other disciplines act a bit presumptuous here. The internist does not take the findings from the ECG serious. The surgeon does not take wound care serious, and so forth. This is completely wrong and is bad, too.” (Surgeon 1, hospital).*

Some respondents suggest family doctors themselves express the frustration and demotivation towards medical students. During their medical clerkship they saw family doctors who incessantly complained about full waiting rooms at an inadequate reward. Thus, medical students obtain a negative impression of the primary care profession and sort it out as option for their future career.

*“(…) If then the self-motivation has decreased, being a family doctor does not mean so much for myself that I do take all these things with me, then it is understandable that they [the students] search for an other job.” (Internist 6, practice).*

## 4. Discussion

The results of the study show that predominantly positive opinions do shape the image of family doctors from the perspective of specialists. Especially the broad medical knowledge is rated positive. The ability for empathy and a farsighted management of care tailored to the individual patient are appraised as important competencies of a family doctor. Perceived negatively is a reduction to the function of a gateway and a growing dissatisfaction among family doctors, which might result in potentially adverse effects on patient care. Underrepresentation of family medicine at the universities as well as negative comments about family doctors by other disciplines in the course of the study was reported as possible negative factors for the family doctor's image. In the opinion of specialists the social prestige of family doctors has changed in the past years. Due to the internet especially younger patients would be better informed and would consult specialists directly. There was no evidence for systematic differences in the opinions, neither between the different specialist groups, nor between rural and urban regions, or male and female specialists. According to previous studies family doctors have the impression that specialists who work in hospitals, have a rather negative image of family doctors compared to specialists practicing in ambulatory care [3]. The present study does not confirm this impression but rather shows an overall more positive picture of family doctors than is assumed by the family doctors themselves without relevant differences between different specialist groups or those working in a hospital or private practice. Amongst other things the specialists in the present study hold the family doctors themselves responsible for negative aspects concerning the image of family doctors. The dissatisfaction among family doctors tarnished their public professional image and warned young academics off. However, some of the respondents questioned the relevance of the traditional profession as family doctor in an age of specialization and mechanization because of the nature of an “all-rounder” not harmonizing with modern ideas. Similar trends are found in studies coming from Switzerland [12, 13]. Also the authors of a Canadian study, where family doctors were qualitatively interviewed, conclude from their results that there is a lack of respect of specialists towards their primary care colleagues [1]. At least at first glance the results of the present study—in which for the first time specialists were asked about this topic—point to a different direction. However, certain expressions and phrases in the present study suggest subliminal negative assessments, too. Here a possible bias due to the method of data collection needs to be taken into account, since the interviews were conducted by a doctoral student of a department of family medicine and health services research.

A possible measure to promote the exchange between family doctors and specialists could be joint training activities such as quality circles. This would help family doctors in recognizing that their image from the perspective of specialists is characterized not as negative as assumed and the mutual respect for the work of the other could grow [14].

Statements concerning the influence of social change are consistent with those of a previous qualitative study with family doctors in Germany [8]. Family doctors also see a reason for the direct consultation of specialists in the fact that particularly younger patients keep themselves informed about medical issues on the internet. In addition, society tends to rate technical diagnostic in specialized practices increasingly higher [14].

As our results show, in the view of the specialists the prestige of family doctors is also strongly influenced by politics. The (impending) shortage of family doctors in Germany currently entails a strong political support for family medicine. At present it cannot be estimated how such measures will change the prestige of family doctors in the eyes of specialists, students, or the public.

Studies in the field of undergraduate education have shown that “badmouthing” of family medicine negatively influences students in their career choice [5, 15, 16]. “Badmouthing” is defined as “unjustified, negative, and slanderous comments by physicians about other physicians in various branches of medicine” [15]. An Australian study on “badmouthing” reports that 12% of Australian medical students had changed their decision to become a family doctor because of negative comments [15]. Also in our study some of the participants report about experiences of “badmouthing” at the university. The lack of representation at the universities might contribute to the fact that the professional image of a family doctor is virtually unknown to some students. As of today only 19 out of the 36 high school sites have at least one institutionalized (part time) professorship [17]. An intensification of teaching and a stronger emphasis on the positive aspects of an activity as family doctor would be a prerequisite in order to gain more students interested in the subject [18].

### 4.1. Strengths and Weaknesses

To our knowledge this is the first qualitative study evaluating the perceptions of specialists regarding the image of family doctors in Germany. A strength of this study is the widely spread sample which comprises different criteria such as specialty, region and hospital, or private practice.

When interpreting the data it must be considered that a tendency toward socially desirable answers from the side of the experts cannot be excluded. Indeed, the specialists were informed prior to the interviews that the interviewer was mandated by the Department of General Practice and Health Services Research to carry out the survey. Also, before any questioning the interviewer stressed the fact that she is a medical student with a neutral position and that the respondents should freely and openly think about the job profile of family doctors.

## 5. Conclusion

Lecturers in medical education and training should become aware of their key role regarding the career choices of young academics and avoid depreciation or upvaluation of particular medical disciplines. In all medical faculties universities professorships for family medicine have to be installed to strengthen education and research in family medicine. Against the background of social processes of transformation, image-enhancing measures would be appropriate, so that the “traditional family doctor's image” in the public (and students) perception will be replaced by a “modern family doctor's image.” New approaches to professional training such as, for example, the “Verbundweiterbildung^plus^” (advanced composite training program) already aim at providing a “modern” image family doctors in Germany [19]. However, politics and medical chambers have to adjust the conditions to foster new forms of employment and cooperation. Taking all these measures will contribute to regain attractiveness for the family doctor's profession as a career choice for young doctors.

## Figures and Tables

**Table 1:** Sample of joining specialists (*N* = 16).

Criteria	Number
Gender	
(i) Female	*n* = 5
(ii) Male	*n* = 11
Location hospital	
(i) University hospital	*n* = 3
(ii) Hospital	*n* = 3
Location private practice	
(i) City (more than 100.000 habitants)	*n* = 4
(ii) Town (20.000–100.000 habitants)	*n* = 4
(iii) Small town (5.000–20.000 habitants)	*n* = 2
Specialties	
(i) Internal medicine	*n* = 6
(ii) Surgery	*n* = 2
(iii) Psychiatry	*n* = 4
(iv) Neurology	*n* = 2
(v) Radiology	*n* = 2

**Table 2:** Positive and negative image aspects.

Main category	Subcategory
	(i) Broadly based medical knowledge
Positive aspects	(ii) Function as gateway
	(iii) Empathy

	(i) “Subliminal negative comments”
Negative aspects	(ii) Frustration of family doctors
	(iii) Old-fashioned image of family doctors

**Table 3:** Social aspects.

Main category	Subcategory
Public reputation	(i) First and continuing contact point for patients
	(ii) Change of the structures (e.g., joint practices)
	(iii) Influence by political activities
	(iv) Influence by the media

Change of patient expectation	(i) Medical information via internet
	(ii) Common desire for technical diagnostics
	(iii) In the cities availability of many specialists
	(iv) Younger patients prefer direct access to specialists

**Table 4:** Influence at university.

Main category	Subcategory
	(i) No lectures
Underrepresentation of family medicine	(ii) Lectures at unfavorable times
	(iii) Experiences with family medicine depend on own initiative of the students

	(i) Negative comments (“badmouthing”)
Image placing	(ii) “Upgrading” of specialized disciplines
	(iii) Frustration among family lecturers
